# Design, characterization and evaluation of a laser-guided focused ultrasound system for preclinical investigations

**DOI:** 10.1186/s12938-019-0656-z

**Published:** 2019-03-28

**Authors:** Pavlos Anastasiadis, Ali Mohammadabadi, Meyer J. Fishman, Jesse A. Smith, Ben A. Nguyen, David S. Hersh, Victor Frenkel

**Affiliations:** 1 Department of Diagnostic Radiology and Nuclear Medicine, University of Maryland School of Medicine, 22 S. Greene St., Baltimore, MD 21201 USA; 2 Marlene and Stewart Greenebaum Comprehensive Cancer Center, University of Maryland School of Medicine, Baltimore, MD USA; 3 Department of Neurosurgery, University of Maryland School of Medicine, Baltimore, MD USA

**Keywords:** Focused ultrasound, Laser targeting, Simulations, Hydrophone measurements, Hydrogel phantoms, Acoustic transmission, Blood–brain barrier, Microbubbles, Microhemorrhage

## Abstract

**Background:**

The clinical applications of transcranial focused ultrasound continue to expand and include ablation as well as drug delivery applications in the brain, where treatments are typically guided by MRI. Although MRI-guided focused ultrasound systems are also preferred for many preclinical investigations, they are expensive to purchase and operate, and require the presence of a nearby imaging center. For many basic mechanistic studies, however, MRI is not required. The purpose of this study was to design, construct, characterize and evaluate a portable, custom, laser-guided focused ultrasound system for noninvasive, transcranial treatments in small rodents.

**Methods:**

The system comprised an off-the-shelf focused ultrasound transducer and amplifier, with a custom cone fabricated for direct coupling of the transducer to the head region. A laser-guidance apparatus was constructed with a 3D stage for accurate positioning to 1 mm. Pressure field simulations were performed to demonstrate the effects of the coupling cone and the sealing membrane, as well as for determining the location of the focus and acoustic transmission across rat skulls over a range of sizes. Hydrophone measurements and exposures in hydrogels were used to assess the accuracy of the simulations. In vivo treatments were performed in rodents for opening the blood–brain barrier and to assess the performance and accuracy of the system. The effects of varying the acoustic pressure, microbubble dose and animal size were evaluated in terms of efficacy and safety of the treatments.

**Results:**

The simulation results were validated by the hydrophone measurements and exposures in the hydrogels. The in vivo treatments demonstrated the ability of the system to open the blood–brain barrier. A higher acoustic pressure was required in larger-sized animals, as predicted by the simulations and transmission measurements. In a particular sized animal, the degree of blood–brain barrier opening, and the safety of the treatments were directly associated with the microbubble dose.

**Conclusion:**

The focused ultrasound system that was developed was found to be a cost-effective alternative to MRI-guided systems as an investigational device that is capable of accurately providing noninvasive, transcranial treatments in rodents.

## Background

The use of therapeutics specifically for the treatment of brain disorders is impeded by the presence of the blood–brain barrier (BBB). The BBB’s primary role is to regulate the exchange of substances between the blood vessels and the underlying tissues, protecting the brain from the entry of potentially harmful compounds [1]. Because the BBB’s protective mechanisms are highly effective, it is a major obstacle in the effort to deliver drugs and other agents systemically to the brain. This has led, in turn, to the development of various techniques including direct intracranial administration of agents [2] or the use of systemically administered agents that can transiently alter the BBB’s permeability [3]. These techniques, however, are either invasive, with associated risks for the patients, or they suffer from poor spatial and temporal control [1].

Focused ultrasound (FUS) is an emerging technology for the treatment of brain disorders [4]. Earlier investigated applications, currently in clinical use, include thermal ablation of uterine fibroids [5], bone metastases [6] and breast cancer [7]. Most recently, FUS was approved by the FDA for the treatment of essential tremor (ET), a debilitating neurological condition involving dysfunctional neural circuits that is normally treated with surgically-implanted deep brain electrodes. The noninvasive MRI-guided FUS (MRgFUS) procedure involves transcranial exposures for the ablation of the ventral intermediate (VIM) nucleus, located within the thalamus [8]. The ability to safely target the VIM nucleus for ET has created the opportunity to apply FUS to other brain-related applications, including enhanced therapeutic delivery to the brain. To date, a number of preclinical studies have reported that MRgFUS can safely and transiently open the BBB, enabling a wide range of therapeutic agents to enter the brain at a targeted location [9, 10], including adult stem cells [11]. Whereas MRgFUS can be very effective for the stated purpose, these preclinical systems are often expensive to purchase and operate. Treatments can also be prohibitively long and are potentially restricted to imaging centers within the research institution where studies are being carried out. While in some cases MRI-guidance is necessary for accurately targeting discrete locations in the brain, often, for general mechanistic studies, it is not required.

In this paper, we present a custom-FUS system for noninvasive, transcranial treatments in small animals. The system is portable, employing laser guidance with a 3D positioning stage for precise targeting of the focused beam without MRI. We designed a custom cone that is filled with degassed water for direct coupling of the transducer to the head of the animal. The degassing setup was also custom-built for the system. Computer simulations were performed to characterize the pressure field generated by the transducer with and without the cone, characterize the location of the focus, and predict transmission losses across the skull over a range of animal sizes. The approximate position and dimensions of the focus were then validated using high-powered, continuous FUS exposures in hydrogel phantoms for the generation of visibly opaque thermal lesions. Exposures were also performed in ex vivo tissue to validate the targeting of the custom, laser guidance apparatus. Finally, proof-of-concept studies were carried out in vivo for opening the BBB in healthy Sprague–Dawley rats. The experiments were designed to confirm the safety and efficacy of the system, as well as its applicability for mechanistic investigations of the process of BBB opening.

## Methods

### System design

**Fig. 1:**
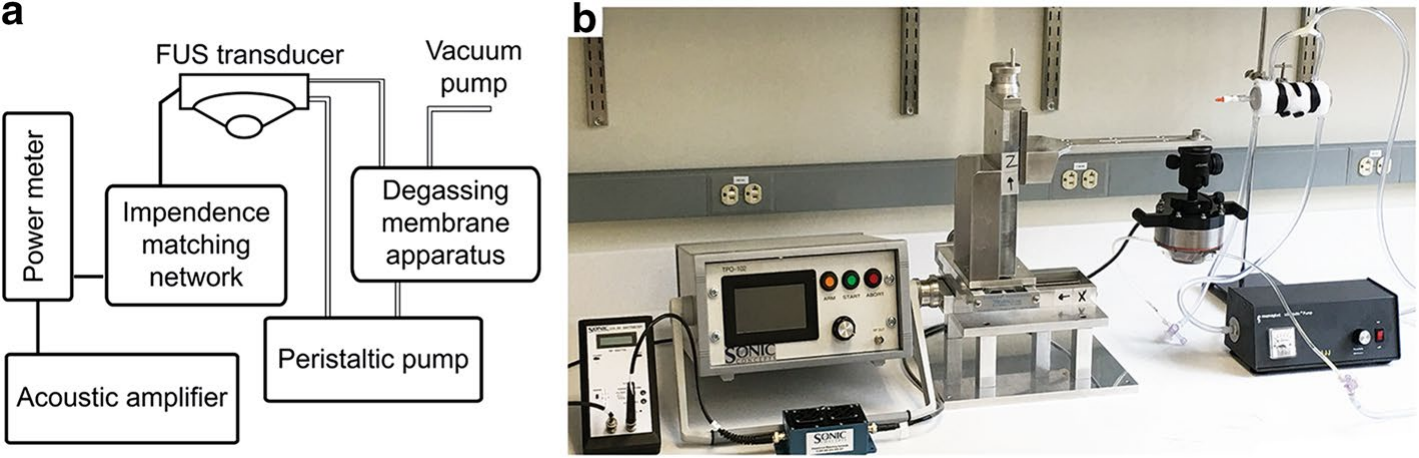
The FUS system: **a** Schematic diagram of the system components; **b** digital image of the system

The major component of the system (see Fig. 1) is a spherical, single-element FUS transducer (Sonic Concepts, Bothell, WA, USA) operating at a center frequency of 500 kHz and a frequency bandwidth ranging from 400 kHz to 600 kHz. The curvature radius and aperture diameter are 63.2 mm and 82 mm, respectively, yielding an F-value of 0.77. The transducer possesses a focal width (− 6 dB) of 2.36 mm and focal length (− 6 dB) of 13.50 mm. A clear plastic cone was designed and constructed to facilitate coupling of the transducer to the target. The height of the cone is 26.6 mm from the exit plane of the transducer housing to the exit plane of the cone. An acoustically transparent (0.05″) silicone membrane (McMaster-Carr, Elmhurst, IL) seals the exit plane of the cone, allowing for direct coupling between the transducer and the target. The membrane can be remotely inflated or deflated to offset the position of the fixed focal distance within the designated treatment region. The cone is filled with water that is degassed using an in-line degassing membrane (PermSelect, Ann Arbor, MI, USA) powered by an external vacuum pump. The transducer is driven by an acoustic amplifier (TPO-102, Sonic Concepts, Bothell WA, USA) via a standard impedance matching system (50 Ω). The amplifier supplies a maximum output power of 150 W. The graphic user interface (GUI) allows for varying the power (at increments of 0.1 W), the pulse width from 10 μs to 1 s (at increments of 1 μs), the pulse repetition frequency from 1 kHz to 0.1 Hz (at increments of 1 kHz), and the treatment duration at increments of 0.1 s. Varying the levels of the different exposure parameters is carried out by selecting the appropriate parameter on the touch screen of the GUI and using a digital dial to increase or decrease its value at their corresponding increments.

A custom positioning system (Velmex, Bloomfield, NY, USA) was assembled using three aluminum alloy axes to allow for independent movement in the *x* (side-to-side), *y* (forward-and-back) and *z* (up-and-down) directions relative to the transducer. Additional custom-designed components manufactured specifically for the system by the company include a 30.5 cm aluminum alloy bracket arm (attached to the vertical *z*-axis) and a 30.5 cm × 30.5 cm platform (Velmex, Bloomfield, NY, USA). Each axis has an independent travel length of 12.7 cm at a resolution of 1 mm. A custom 3D-printed adapter (Makexyz, Austin, TX, USA) was designed to connect the transducer to a ball-joint camera mount (Sinvitron, Hong Kong, China), with the latter attaching to the bracket arm. The combined axes and the ball-joint mount allow for a total of five degrees of freedom in movement, rendering the positioning system sufficiently flexible for a variety of treatment applications.

**Fig. 2:**
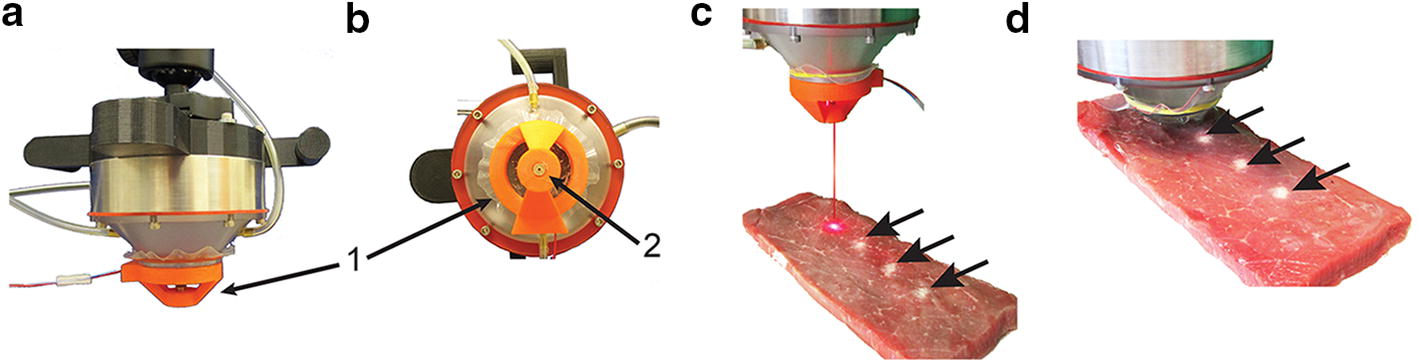
Laser-based targeting of the FUS treatments: **a** Laser-based apparatus labeled “1”. **b** Underside view of the exit aperture of the laser labeled “2”. **c** Ablative exposures in beef muscle tissue were used to demonstrate the accuracy of the targeting, where three thermal lesions (arrows) are visible. The distance between the individual treatment locations was 6 mm. The thermal lesions (arrows) were created using continuous wave exposures at 20 W for a treatment duration of 30 s. Treatment planning and targeting with the laser is shown for a fourth location. **d** The targeting apparatus has been removed and the transducer brought into position for the treatment of the fourth lesion

A laser-based apparatus was designed for targeting the exposures in the (*x, y*)-plane. The apparatus was custom-made with a 3D printer (LulzBot TAZ 5, Aleph Objects, CO) for a single off-the-shelf laser (Fig. 2a, b). The 650 nm dot laser (DIYMall, Shenzhen, China) was operated at 5 V. The apparatus was designed such that it can be placed onto the exit plane of the cone using ultrasound coupling gel. Once targeting is determined during treatment planning, the apparatus is then removed. Demonstration of the suitability and accuracy of this procedure was carried out using thermal ablations in ex vivo tissue samples (Fig. 2c, d) [12]. The laser apparatus was used for the in vivo treatments (below), employing stereotactic coordinates for targeting specific regions within the brain.

### Transducer calibration

A radiation force balance-based power meter (Ohmic, Easton, MD) was used for calibrating the FUS transducer. Continuous exposures were carried out at 5 W, 7 W, 12 W, 15 W, 17 W and 20 W (n = 5 for each power) as indicated on the GUI of the amplifier. Measurements were carried out in degassed water to minimize the effects of acoustic cavitation. Exposures were maintained until the output power stabilized. An average efficiency (measured power/applied power × 100) of 86% was observed over the range of powers that were evaluated. All powers cited for the remainder of the study were those calibrated at this level of efficiency.

### Simulations of the acoustic pressure fields

Computer simulations were carried out to characterize the acoustic pressure fields generated by the FUS transducer. These were performed under four conditions: in free-field for the transducer alone; in free-field for the transducer with the cone but without the membrane; and for the transducer with the cone, including the acoustically transparent membrane, both inflated and non-inflated. The geometry of the bolus was simulated as an arc with a height corresponding to the height of the inflated membrane in the experimental setup for the measurements carried out to validate the simulations (see below). These four conditions were considered for two reasons: (i) to determine if the presence of the cone affected the ultrasound field, and (ii) to demonstrate the importance and advantage of using degassed water encased within the cone that was sealed by the membrane.

The simulations were performed using a two-dimensional axisymmetric model developed in COMSOL version 5.3 (COMSOL, Burlington, MA). COMSOL solves the non-linear wave propagation equation to calculate the magnitudes of the acoustic pressure fields. With the commonly used assumption that the pressure generated by the transducer can be represented as a simple harmonic sinusoidal wave, the Helmholtz equation [13] can be reduced as:

1 $$\nabla \cdot \left[ { - \frac{1}{{\rho_{0} }}\left( {\nabla p} \right)} \right] - \frac{{\omega^{2} }}{{\rho_{0}^{2} }}p = 0$$

where *p* is the pressure (Pa), *ρ* is the density of the medium (kg/m^3^), *ω* is the angular frequency (rad/s) and *c* is the speed of sound (m/s). The simulations were carried out at the center frequency of the transducer at 500 kHz in continuous mode. The material properties of the different components of the system were obtained from the COMSOL materials library and additional sources [14, 15] and appear in Table 1. These include the cone, made of polyvinyl chloride (PVC) and the acoustically transparent membrane, made of silicone rubber.

**Table 1:** Properties of the materials used in the simulations of the device design

	Water (8% air)	Water (2% air)	PVC	Silicone rubber
Density (kg/m^3^)	998	998	1760	1250
Speed of sound (m/s)	1498	1498	1060	1460
Attenuation (dB/cm)	0.0351	0.0092	1.1200	0.0875

### Localization of the focus

In order to verify the position of the focus of the FUS transducer, exposures were carried out in ultrasound hydrogel phantoms. The phantoms were prepared as previously described [12]. Briefly, bovine serum albumin (BSA) was dissolved in a polyacrylamide hydrogel solution at a percentage of 7% (w/w) and allowed to set. Phantom samples (30 mm in height, width and length) were treated at their approximate geometrical center, based on our simulation results. The exit plane of the cone was positioned just above the surface of the phantoms, with the bolus deflated. Degassed ultrasound coupling gel was added between the bolus and phantom. The bolus was then inflated remotely with a syringe (filled with degassed water) so that the bolus was in direct contact with the phantom with a thin layer of gel between them. Ultrasound exposures were carried out at 80 W in continuous mode for a duration of 20 s (n = 6) to create a thermal lesion. Scaled images of the lesions within the phantoms were captured with a digital camera. Lighting provided behind the phantoms was used to delineate the lesions and determine their location relative to the exit plane of the cone.

### Hydrophone measurements of the acoustic pressure fields

In order to validate the simulations, we carried out hydrophone measurements using a custom setup. Measurements were carried out in a water tank (51 cm × 27 cm × 32 cm) filled with deionized water. A needle hydrophone (Onda, Sunnyvale, CA, USA) was positioned in the approximate region of the focus, across from the FUS transducer. The hydrophone was held in position using a custom holder (Solidworks, Waltham, MA, USA) and fabricated using a 3D printer (LulzBot TAZ 5, Aleph Objects, CO).Determining the exact position of the focus required an iterative process, where the transducer was moved in all three dimensions (*x*, *y* and *z*) to maximize the measured voltage. Measurements were recorded using a digital oscilloscope (Tektronix, Beaverton, OR, USA) at 0.5 W, 0.7 W, 1.0 W, 1.2 W and 1.5 W. The ultrasound exposures were carried out in continuous mode (n = 5 for each power). As in the simulations, measurements were recorded in free field for the transducer alone, the transducer with the cone, and the transducer with the cone and acoustically transparent membrane both inflated and non-inflated.

### Simulating transmission across the skulls

In order to investigate the effect of the skull on our treatments in rats, we first carried out simulations using our COMSOL model (described above). In our model, we positioned a rectangular section of skull in the immediate pre-focal region, which was representative of in vivo conditions. The simulation model incorporated the speed of sound, density and attenuation coefficient for skull bone [16] (Table 2). The peak acoustic pressure at the focus was determined with and without the presence of the skull at the center frequency of the transducer (500 kHz). Values of the acoustic pressures were simulated over a weight range from 80 to 675 g, based on the weight range of the rats from whom skulls were scanned with computed tomography (CT) for thickness measurements. The pressures were normalized to values without skulls.

**Table 2:** Acoustic properties of water and bone in the simulations of skull transmission

Tissue type	Density (kg/m^3^)	Speed of sound (m/s)	Attenuation (Np/m/MHz)
Water	1000	1482.3	0.025
Skull bone	1700	3183	164

### Measuring transmission across the skulls

**Fig. 3:**
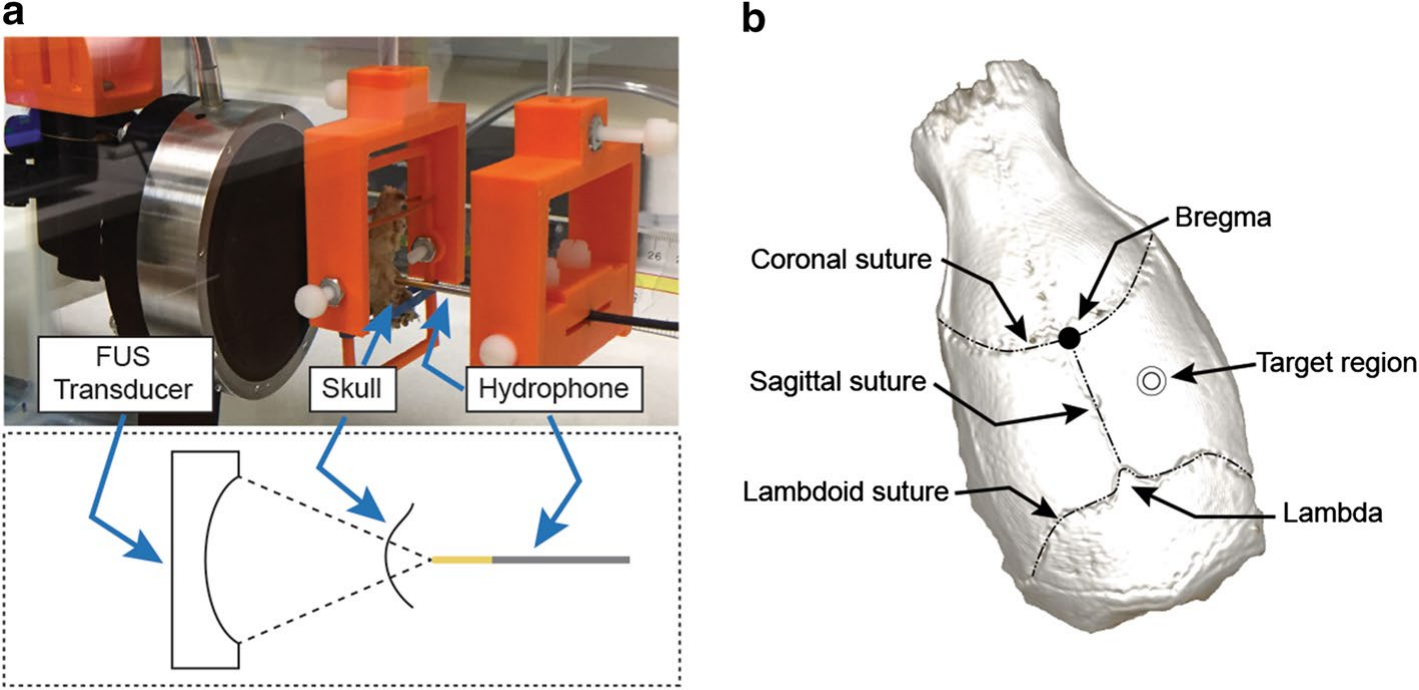
Attenuation measurements in the rat skull: **a** Experimental setup for measuring the acoustic attenuation in the rat skulls. The FUS transducer, skull holder, and the needle hydrophone are aligned inside the water tank. **b** Micro-computed tomography image depicting the anatomical landmarks on the rat skullcap, indicating where the measurements were taken for the rat skull in ‘**a**’

The hydrophone setup (described above) was used to determine the acoustic transmission across the skulls of the rats. Skulls were harvested from a range of different sized female, Sprague–Dawley rats (67.4 g to 630 g; n = 3 per weight). Rats were euthanized, and their skulls removed and cleared from residual brain tissue with a mild enzymatic detergent. The skulls were then rinsed and degassed in a custom vacuum chamber prior to measurements [17]. A custom holder was designed for the skulls (Solidworks, Waltham, MA, USA) and fabricated using a 3D printer (LulzBot TAZ 5, Aleph Objects, CO). Measurements were carried out in the same water tank as described above (Fig. 3a).

For the baseline reference measurements without the presence of a skull, the acoustic attenuation was measured for the water path, between the transducer and the hydrophone. Measurements were recorded on a digital oscilloscope (described above). At 1 and 2 Watts, consistent measurements were observed, however, below the threshold of acoustic cavitation in the beam path. The FUS exposures were carried out in continuous mode (n = 5 at each power). For measurements with the skulls, they were positioned such that the parietal bone (Fig. 3b) was located just in front of the hydrophone tip (as would occur during an in vivo treatment), half way between the bregma and the lambda, and half way between the sagittal suture and the outer edge of the skull.

### In vivo treatments for opening the BBB

**Fig. 4:**
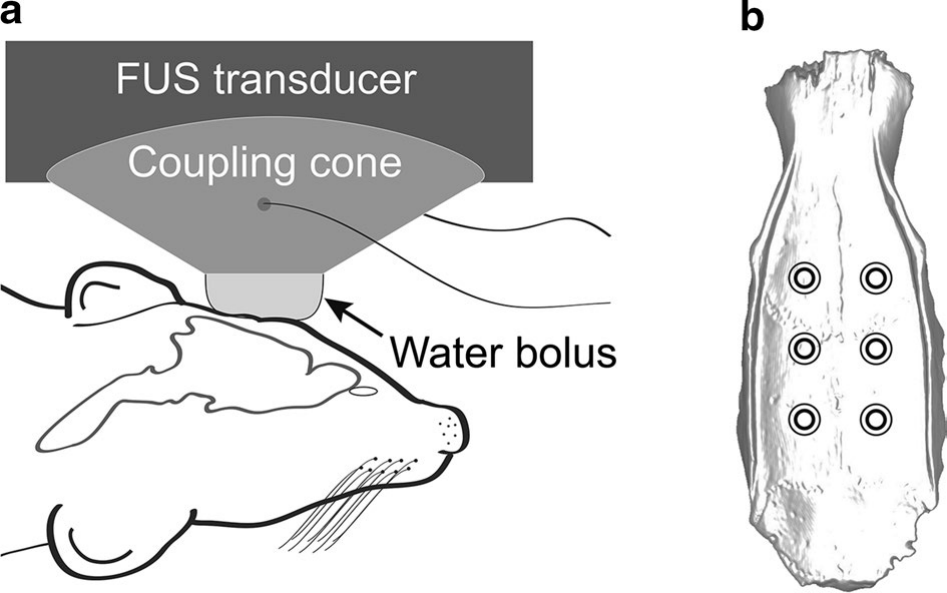
Schematic representation of the in vivo transcranial FUS treatments: **a** The FUS transducer is positioned on the animal’s head and coupled via a cone and inflated acoustically transparent membrane (bolus) containing degassed water. **b** Raster pattern of the six individual treatments (circles) that were given in succession immediately following the administration of the MBs for the in vivo treatments

Animal experiments were carried out according to an approved protocol by the University of Maryland School of Medicine Institutional Animal Care and Use Committee (IACUC). For the treatments, female Sprague–Dawley rats were used over a range of weights (see below). All procedures were carried out under anesthesia using inhalation isoflurane (~ 2.5%, O_2_ L/h). The animals were shaved using electric clippers, followed by a brief application of a depilatory cream to remove all residual hair for maximal coupling. The animals were then positioned in a stereotactic frame. The FUS transducer was positioned over the head of the animal using the laser guidance apparatus. Once the location of the initial treatment location was set, the laser apparatus was removed, and the transducer coupled to the animal’s head using ultrasound gel (Fig. 4a).

With the transducer in position, animals were injected via a tail-vein catheter with a dose of commercially obtained monodisperse (4–5 μm) microbubbles (MBs) (Advanced Microbubbles Laboratories, Boulder, CO, USA). MBs were diluted with normal saline to a final volume of 400 μL prior to infusion. Immediately following a single injection of MBs, the FUS treatments were started. For the first two sets of animals, six individual locations were treated in a 2 × 3 raster pattern (Fig. 4b), covering the entire head with a lateral spacing of 2.5 mm, the approximate radial diameter (− 6 dB) of the focus. Exposures were carried out for 2 min per location using a 10% duty cycle and pulse repetition frequency of 10 Hz (10 ms ON; 90 ms OFF). The dose of the MBs was 25 µL and 50 µL for the group of smaller (113 g to 122 g) and larger (234 g to 250 g) animals, respectively. Acoustic pressures of 0.1 MPa, 0.2 MPa, 0.3 MPa, 0.4 MPa and 0.5 MPa were evaluated in each group of animals, corresponding to powers of 0.9 W, 1.0 W, 1.4 W, 1.7 W and 2.0 W, respectively. The objective of the treatments was to determine the threshold acoustic pressure for opening the BBB in each size group of animals (n = 3).

Once the treatments were finished, the group of smaller animals (113 g to 122 g) was injected with a single dose of 4 mL/kg of Evans blue dye (EBD) via the tail-vein at a concentration of 4% in 0.9% phosphate buffered saline (PBS, pH 7.4) [18]. Following a 30 min period to enable uniform circulation of the EBD [18], the animals were perfused with 30 mL PBS; (pH 7.4) followed by 30 mL of a 4% paraformaldehyde (PFH) solution in PBS. The animals were then euthanized, and their brains removed and sectioned at 1 mm slices using a brain matrix slicer (Zivic Instruments, Pittsburgh, PA, USA). Observations were carried out for the presence of EBD, as a positive indicator of BBB opening. Digital images were captured of each slice on both sides.

FUS exposures for BBB opening were carried out in a second group of larger animals (234 g to 250 g). BBB opening was validated by MRI as previously described [11]. Instead of EBD, a gadolinium-based contrast agent was injected systemically (0.8 mL/kg; Omniscan, GE Healthcare, Princeton, NJ). The animals were then scanned by T1-weighted (T1w) images for the presence of the contrast agent, which appears as a hyperintense signal.

All in vivo MRI scans were performed using a Bruker BioSpec 70/30USR Avance III 7T horizontal bore MR scanner (Bruker, Billerica, MA, USA) equipped with a BGA12S gradient system and interfaced to a Bruker Paravision 6.1. A Bruker four-element [1] H RF surface coil array was used as the receiver and a Bruker 72 mm linear RF volume coil as the transmitter. T2-weighted (T2w) MR images in the axial and coronal view were obtained by a two-dimensional rapid acquisition with relaxation enhancement (RARE) sequence with repetition time (TR) of 2500 ms and 3500 ms, respectively. The effective echo time (TE_eff_) for the axial view was 24 ms and 40 ms for the coronal view. The field of view was 45 pixels × 35 pixels for the axial view and 35 pixels × 35 pixels for the coronal view. For T1w MR image acquisitions following the administration of gadolinium contrast, we used the RARE sequence with a TR of 300 ms and TE of 8 ms in the coronal view. In all cases the slice thickness was set at 1 mm.

A third set of animals (207 g to 245 g) was treated using three different MB doses (100 µL, 50 µL and 5 µL). Evaluation of BBB opening was carried out by T1w contrast-enhanced MR imaging (described above). Two individual locations were treated at a spacing of 4 mm along the anterior–posterior axis in the left hemisphere at 0.3 MPa for 2 min each. The contralateral right hemisphere did not receive treatment and was used as an internal control. Following treatments, animals were scanned by MRI, employing three separate scanning modes: T2w (for observations of edema), T2* (for observations of microhemorrhage) and T1w (for observations of BBB opening, following the administration of contrast).

Immediately following the MRI scans, animals in the last group were perfused and euthanized, and their brains removed. These were fixed, sectioned, mounted on glass slides and stained with hematoxylin and eosin (H&E) for histological analysis. Observations were carried out with a light microscope at 10× and 20× magnification and representative images were captured using a digital slide scanner (Keyence, Itasca, IL, USA). Observations of the section were carried out for microhemorrhage in the form of red blood cell extravasation, as well as other manifestations of structural damage as previously described by us [11, 19].

## Results

### Localization of the focus

**Fig. 5:**
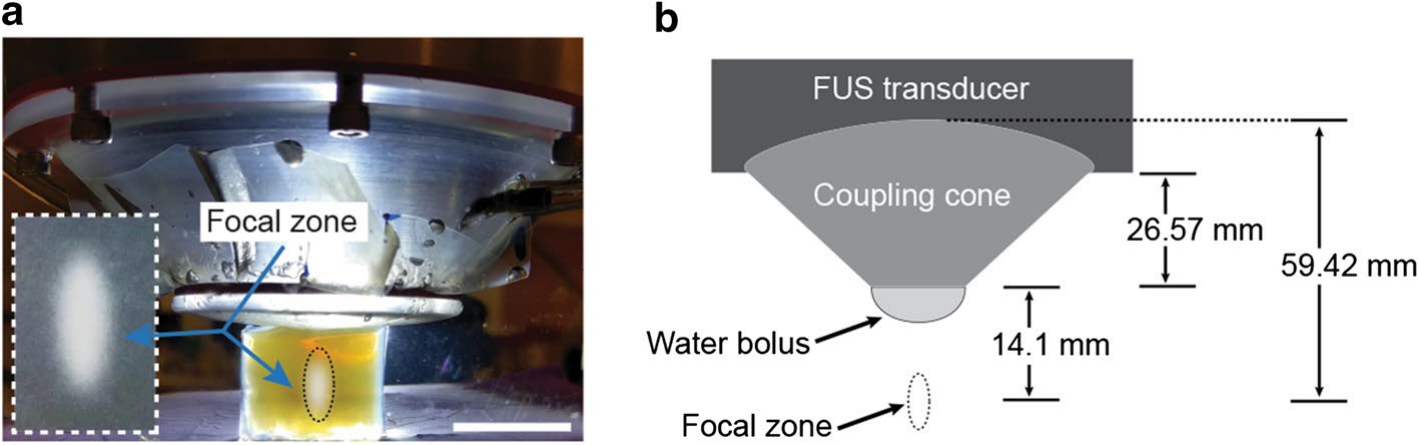
FUS transducer, coupling cone and water bolus: **b** Dimensions of the components, indicating the position of the focus. **a** Digital image of a thermal ablative lesion generated in a polyacrylamide phantom

The FUS exposures in the hydrogel phantoms were found to generate sufficiently high heat to denature the albumin, which becomes optically opaque and visible to the naked eye. Using the scaled images, the mean distance from the center of the lesions to the exit plane of the cone was found to be 13.97 ± 0.29 mm (n = 6). A representative image of the setup and a thermal lesion appear in Fig. 5.

### Simulations of the acoustic pressure fields

**Fig. 6:**
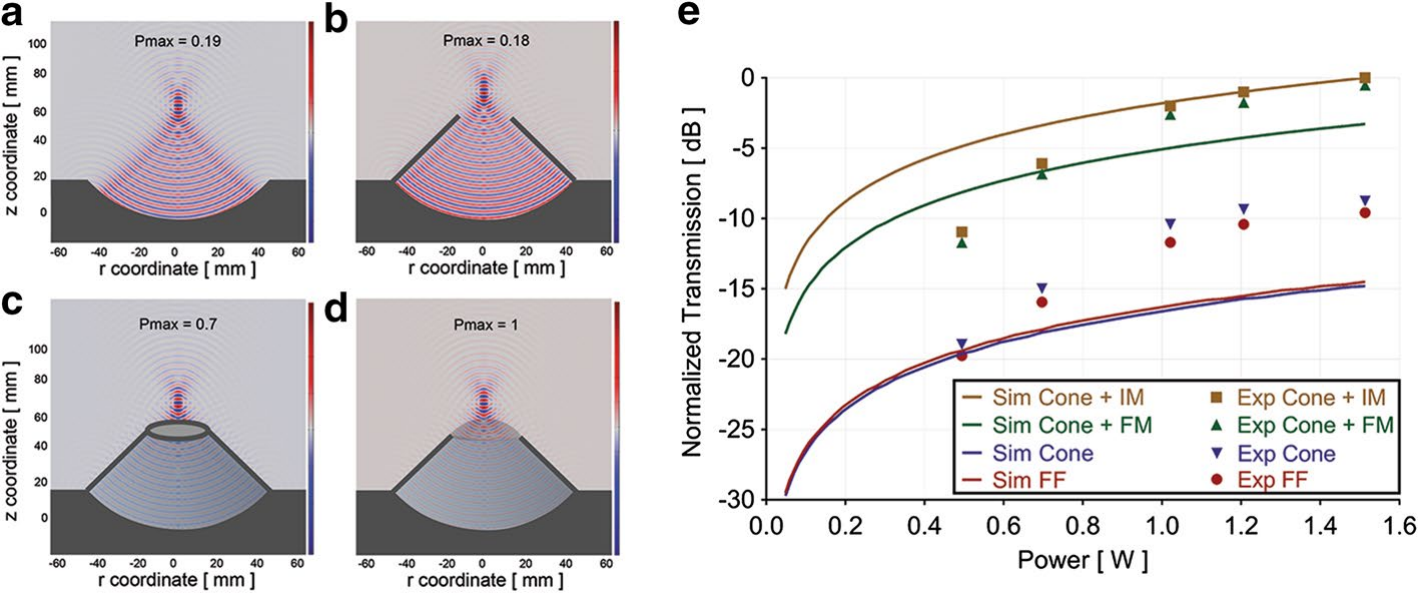
Pressure field simulations: **a** Transducer alone; **b** Transducer with cone; **c** Transducer with cone and membrane uninflated; **d** Transducer with cone and membrane inflated. Output pressure fields in **a**, **b** and **c** were normalized to that of **d**, where the maximal pressure (P_max_) at the focus was found. **e** Comparison of the simulations (Sim) and hydrophone measurements (Exp): transducer alone (FF); transducer with cone (Cone); transducer with cone and membrane non-inflated (FM); transducer with cone and membrane inflated (IM). Values represent normalized acoustic pressures at the focus to those with the inflated membrane, for both the simulations and the hydrophone measurements. All values, simulated and measured, were normalized to that occurring at the maximum power of 1.5 W with the inflated membrane

The position of the focus was determined according to the location of the peak pressure. This was found to occur at 14.1 mm from the exit plane of the cone and was the same for all four conditions evaluated (Fig. 6). The highest of these pressures was with the cone and inflated membrane. The results for the other three conditions were normalized to this value. The acoustic pressure at the focus for free field conditions (Fig. 6a) was found to be 19% of that with the inflated membrane (Fig. 6d). With the cone only, the peak pressure was 18% (Fig. 6b). Simulations with the cone and non-inflated membrane showed the acoustic pressure at the focus to be 70% of that with the cone and membrane inflated (Fig. 6c).

### Hydrophone measurements of the acoustic pressure fields

As for the simulations, measurements with the inflated membrane were used to normalize the other three sets of measurements at each applied power. The measurements were generally found to be in accordance with the simulations when using the cone and membrane, both inflated and uninflated. Without the cone and/or membrane, disparities between the simulations and measurements were relatively greater. The results of the simulations and measurements appear in Fig. 6e.

### Transmission simulations across the skulls

**Fig. 7:**
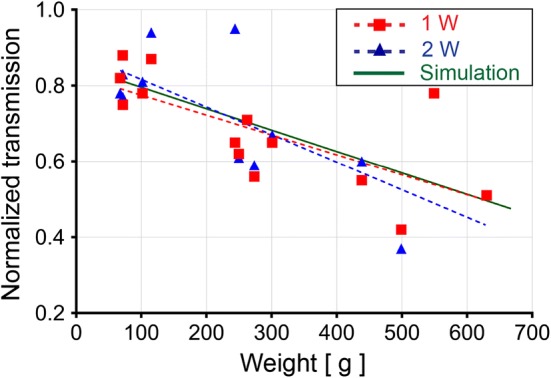
FUS transmission across the skulls: Simulations of FUS transmission across the skulls of rats over a size range of 80 g to 675 g. Measured transmission of FUS across the rat skulls over a size range of 67.4 g to 630 g. Measurements were carried out for continuous FUS exposures at 1 and 2 W

We employed our simulations to determine the relative changes in transmission of ultrasound energy across the rat skull that will occur over the range of animal sizes that are typically used in our studies. In preliminary experiments, we determined the relationship between the size (e.g., weight) of a rat and the thickness of its skull using a small animal CT scanner in order to measure the skull thickness (data not shown). The simulations showed the transmission to be proportionally lower as the size of the rats increased (Fig. 7).

### Transmission measurements across the skulls

Hydrophone measurements were carried out with and without skulls positioned in the pre-focal region at 1 W and 2 W. Similar to the results of the simulations, an inverse relationship was found between the weight of the animals from which the skulls were harvested and the measured transmission. The transmission values were normalized to the measurements taken without a skull. The results, combined with the simulations, appear in Fig. 7.

### In vivo treatments

**Fig. 8:**
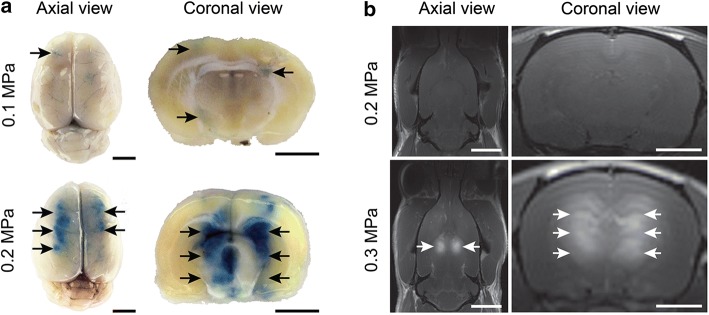
Representative brain images of animals treated for BBB opening. **a** The group of smaller animals treated at 0.1 MPa (top) and 0.2 MPa (bottom) followed by the systemic administration of EBD. Arrows indicate the locations of EBD (blue) signal in whole brains and brain sections. **b** The group of larger animals treated at 0.2 MPa (top) and 0.3 MPa (bottom) followed by systemic administration of gadolinium contrast. Arrows indicate the locations of contrast (hyperintense signal) in T1w MR coronal and axial images. Scale bars in the axial (left) and coronal (right) images correspond to 5 mm and 2.5 mm, respectively

In the first two sets of animals, a range of acoustic pressures was evaluated in order to determine the acoustic pressure threshold for opening the BBB for each animal size. Validation of BBB opening was determined by post-treatment visualization of either EBD or gadolinium contrast in the brain [11]. In the group of smaller animals, the acoustic pressure threshold for BBB opening was found to be 0.2 MPa (Fig. 8a). In the group of larger animals, the acoustic pressure threshold was 0.3 MPa (Fig. 8b).

**Fig. 9:**
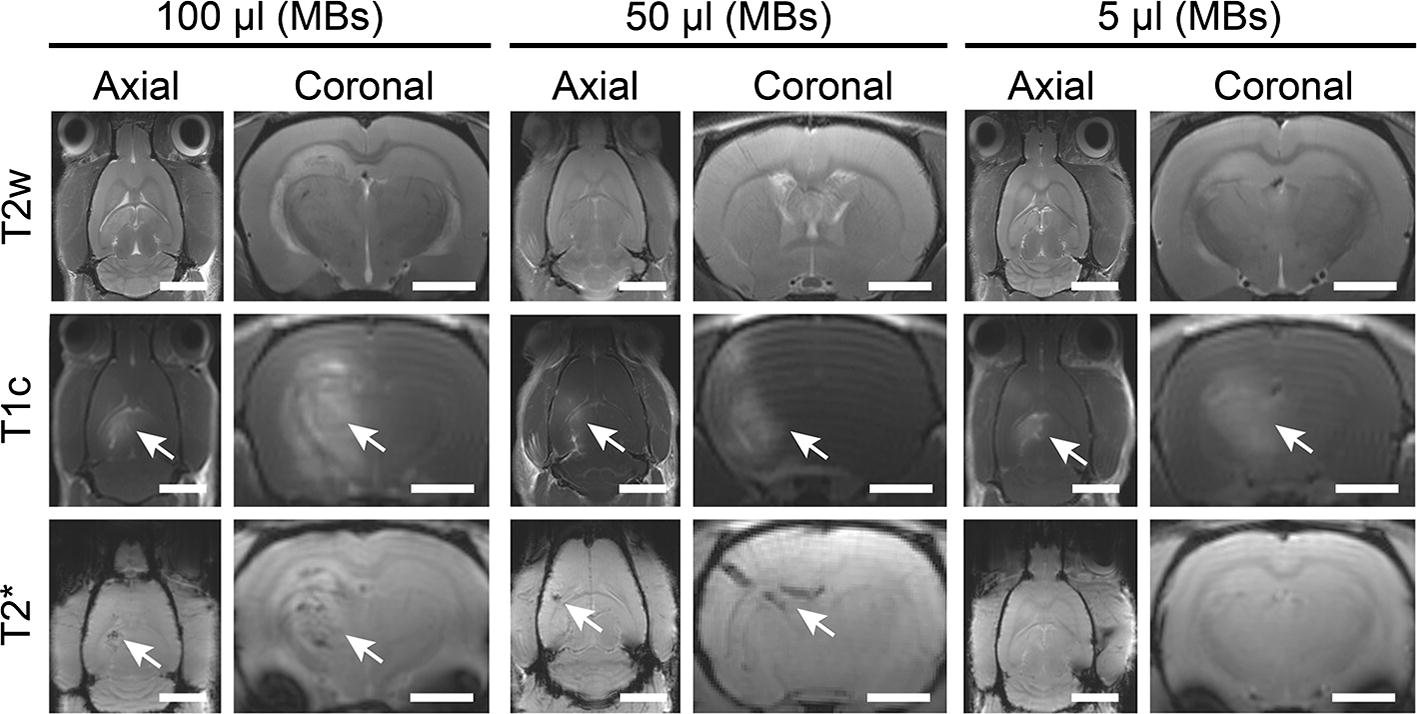
Representative axial and coronal MR images of animals treated for BBB opening at various MB doses: 100 µL, 50 µL and 5 µL. MR images reveal BBB opening (T1c hyperintensity) and microhemorrhage (T2* hypointensity). T2 images for inflammation were inconclusive. Scale bars for the axial and coronal images correspond to 5 mm and 2.5 mm, respectively

**Fig. 10:**
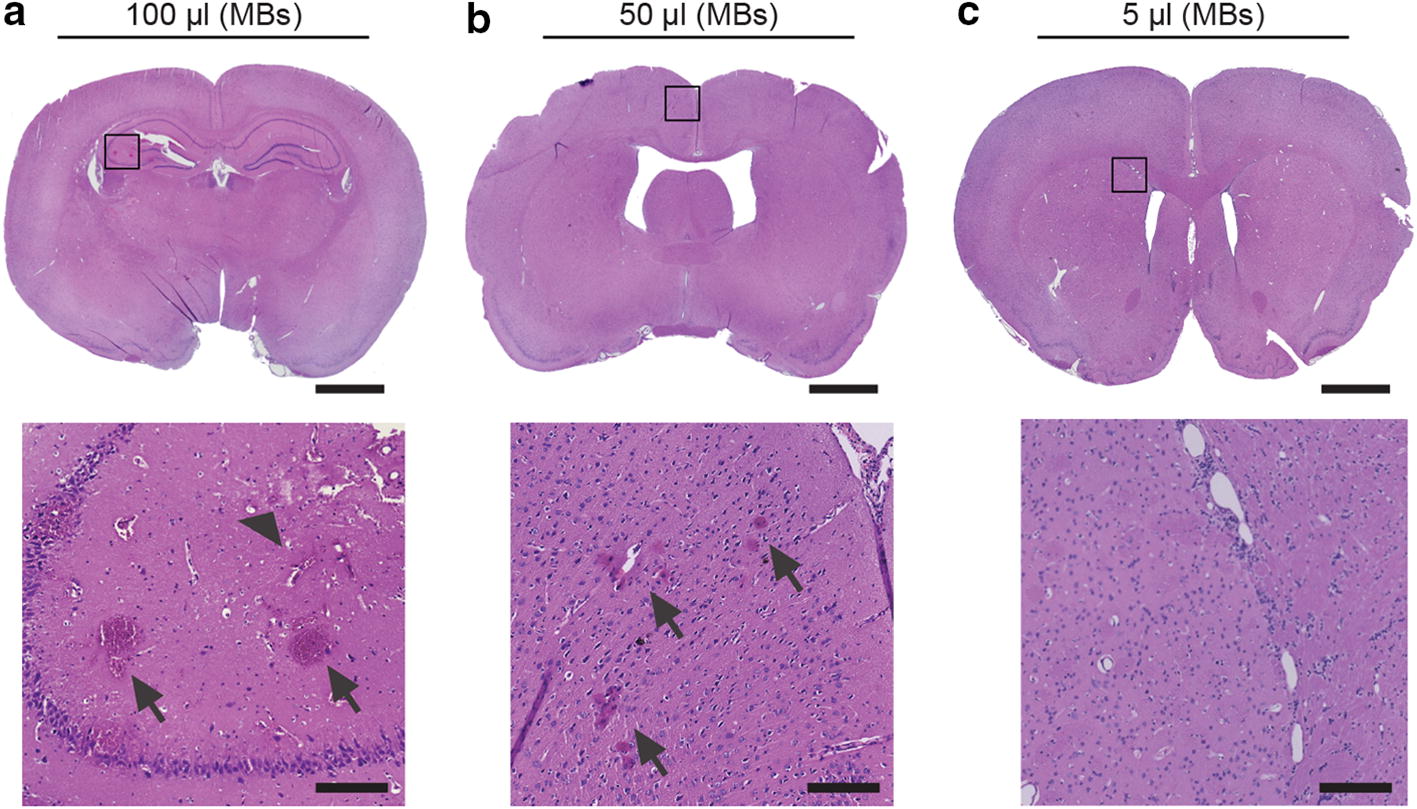
Representative histopathology images of animals treated for BBB opening at various MB doses: 100 µL (**a**), 50 µL (**b**) and 5 µL (**c**). Microhemorrhage (arrows) and structural damage (arrowhead) are noted in higher magnification insets, located directly below each respective image. Scale bars in the whole brains and insets correspond to 2 mm and 200 μm, respectively

In the third set of animals, BBB opening was assessed using T1w MR imaging at each of the three MB doses evaluated (100 µL, 50 µL and 5 µL). BBB opening was greatest at the MB dose of 100 µL, where a large, hyperintense region was observed that covered almost the entire left hemisphere. Microhemorrhage was evident as seen in hypointense signals in the T2* MR images. Less pronounced T1 enhancement was observed at the MB dose of 50 µL. T2* hypointensity was also less evident, indicating a reduction in microvasculature damage. At a MB concentration of 5 µL, T1 hyperintensity was further reduced. However, no T2* hypointensity was observed (Fig. 9). At the 5 µL dose, histological sections showed no evidence of structural damage to the treated brains. Microhemorrhage was observed at 50 µL and found to be more pronounced at 100 µL (Fig. 10).

## Discussion

In this paper, we describe the development of an effective FUS system for noninvasive transcranial treatments in small animal models. Typically, these treatments are carried out using expensive and comparatively hard to access MR-guided systems [20]. However, for many of the investigations that will be required to develop and optimize these treatments in order to expand their application into the clinical realm, we have shown that this can be done without MRI. At relatively little cost, the system was put together using an off-the-shelf transducer and amplifier with other parts, either purchased or produced using an in-house 3D printer. This included a simple laser guidance apparatus that we validated and demonstrated to be accurate, a custom stage with five degrees of freedom and a standard stereotactic frame. Once the system was built and customized, we employed well-established methods and procedures to characterize it, using simulations, measurements with an acoustic hydrophone and exposures in a tissue-mimicking phantom.

Simulations of the location of the focus were validated using the experiments in the hydrogel phantoms, where thermal lesions were generated and their position measured relative to that of the transducer. The disparity between the simulations and measured results was less than 1%. Regarding the simulations of the acoustic pressure at the focus, these were highest with the cone and inflated membrane. These results were not surprising seeing that this configuration ensured that there was degassed water in the entire beam path from the face of the transducer to the focus. In free field the acoustic pressure at the focus was only 19% of that with the inflated membrane, indicating the effect of having non-degassed water in the beam path, where acoustic cavitation can occur. Cavitating bubbles will not only absorb acoustic energy but under specific conditions, also reflect the transmitting beam. Both these factors will contribute to energy loss at the focus [21]. The acoustic pressure with the cone was similar (18%) to that in free field, indicating no effects of the presence of the cone. With a deflated membrane, the pressure was 30% lower than with the membrane inflated, further demonstrating the effects of non-degassed water, which was present at the focus.

Overall, the device design was found to be efficient for transmitting the ultrasound energy and localizing it at the focus. The results of the simulations were more or less in accordance with the hydrophone measurements for the cone with the membrane, both inflated and uninflated. Relatively larger disparities between measured and simulated values were observed for conditions without the cone or with the cone without the membrane. This was most likely due to the inability to accurately simulate the effects of the non-degassed water on the propagating beam compared to degassed water, where interactions of ultrasound energy and the presence of stabilized bubbles is considered a highly stochastic phenomenon. The simulated attenuation of our non-degassed water was apparently greater than it was in the measurement tank, which would explain the simulated values being lower than the measured ones. Once the system was fully characterized, we then carried out experiments related to the in vivo application of the FUS exposures.

The vertebrate skull can have a direct effect on the transmission of ultrasound energy from an external transducer into the brain [17, 22]. This is due to bone having a considerably larger acoustic absorption coefficient than typical soft tissues [23], as well as the impedance mismatch that occurs when ultrasound energy propagates across interfaces (e.g., skull bone to soft brain tissue) with relatively large disparities in acoustic impedance [24, 25]. Whereas the former of these factors will directly affect the transmission of ultrasound energy, the latter will generate reflections and hence affect transmission indirectly.

Investigations were carried out to study the transmission of ultrasound energy through the skull, where this had previously been described in clinical applications [26, 27]. A thicker skull will have greater attenuation, proportional to its thickness. Larger animals of the same species will have larger and proportionally thicker skulls, as was found in experimental rodent models in FUS studies [17, 22]. A thicker skull will generate greater acoustic energy loss in the form of absorption, reflection and scattering of the ultrasound waves. Absorption of ultrasound energy is also dependent on the frequency of the ultrasound wave, where greater absorption will occur at higher frequencies [17]. The overall objective was to predict the relationship between the size of the animals and the relative transmission of ultrasound energy through the skulls of the rats. The motivation for this was to effectively translate our results in animals of one particular size range to those of another range without the need to determine this empirically. The inverse relationship between the size of the animals and the transmission across their skulls that we observed was consistent with the findings of a previous study [17]. This was observed in the simulations and was found to correlate well with the hydrophone measurements.

The last set of experiments involved in vivo treatments in rats that were designed specifically for opening the blood–brain barrier (BBB). In earlier work, we had shown that this procedure could be used to enable the targeted delivery of neural progenitor cells using an MRI-guided FUS system [11]. The procedure involves the administration of gas-filled microbubbles (MBs; e.g., ultrasound contrast agents) administered systemically just prior to pulsed FUS exposures. The varying pressure field of the FUS causes the MBs to oscillate, generating mechanical forces in the microvasculature at the site of treatment. These forces can alter the structural integrity of the tight junctions between endothelial cells, resulting in transient changes in BBB permeability [28]. This procedure is currently being evaluated for enhancing the delivery of chemotherapeutic agents to the brain in clinical trials at our institution for the treatment of invasive brain tumors [29].

To provide proof-of-concept evidence of the utility and sensitivity of the FUS system, we demonstrated that we could safely and accurately open the BBB in live animals. This particular application can be characterized in terms of the threshold acoustic pressure required to activate the MBs for inducing the BBB permeability. The acoustic pressure thresholds were determined by carrying out the treatments in vivo and observing a BBB-impermeable dye and MRI contrast agent in the brains of the animals. The results of these treatments showed that the larger animals required a relatively higher acoustic pressure in order to reach the threshold for opening the BBB, apparently because of their thicker skulls [17, 22].

Additional experiments were performed to further demonstrate the utility and sensitivity of our system. Investigations demonstrated the effect of varying the MB dose. As previously shown, a higher MB dose was observed to generate greater BBB opening at the same acoustic pressure [30, 31]. Above a threshold pressure, however, a greater MB dose also created more structural damage in the tissue in the form of microhemorrhages. Edema, as seen in T2w MR images, also appeared to be greater at the higher doses, but the pattern was less clear over the range of animals treated. Typically, more time post-treatment is needed to accurately employ T2w MR imaging for this purpose. These results are in accordance with previous studies [30, 31]. Only at the lowest MB concentration was no damage observed, indicating the required conditions for safe BBB opening in rats of this size. T2* results indicating microhemorrhage were supported by histopathological analysis with evidence of red blood cell extravasation in these tissues.

## Conclusions

The system we developed was found to be safe and effective for the treatment of small animals. By thorough investigation and validation of the location of the focus, the ability to predict in situ acoustic pressures based on a comprehensive understanding of the factors that influence them in our experimental system, and with an accurate and effective positioning system, including laser guidance, we demonstrated that we could safely and accurately provide treatments without the need for external imaging systems. These results will facilitate various types of treatments (e.g., for opening the BBB or for neuromodulation) over a range of animal sizes, without having to empirically optimize the exposures each time an animal of a different size is treated. Our system, in addition to being useful for treating small animals, also provides the basis for future hand-held devices that could be used clinically at the bedside. Such devices have been proposed for both FUS-mediated neuromodulation in superficial regions of the brain, as well as for improved thrombolysis in the setting of deep vein thrombosis [32, 33].

## Abbreviations

BBB: blood–brain barrier
FUS: focused ultrasound
MB: microbubbles
ET: essential tremor
MRgFUS: MRI-guided focused ultrasound
VIM: ventricular intermediate nucleus
3D: 3-dimensional
PVC: polyvinyl chloride
BSA: bovine serum albumin
MBs: microbubbles
EBD: Evans blue dye
PBS: phosphate buffered saline
T1w: T1-weighted
T2w: T2-weighted
MPa: mega pascal
H&E: hematoxylin and eosin
dB: decibels
CT: computed tomography
DVT: deep vein thrombosis
tPA: tissue plasminogen activator
